# Effective Detection of Human Leukocyte Antigen Risk Alleles in Celiac Disease Using Tag Single Nucleotide Polymorphisms

**DOI:** 10.1371/journal.pone.0002270

**Published:** 2008-05-28

**Authors:** Alienke J. Monsuur, Paul I. W. de Bakker, Alexandra Zhernakova, Dalila Pinto, Willem Verduijn, Jihane Romanos, Renata Auricchio, Ana Lopez, David A. van Heel, J. Bart A Crusius, Cisca Wijmenga

**Affiliations:** 1 Department of Medical Genetics, University Medical Centre Utrecht, Utrecht, The Netherlands; 2 Broad Institute of Harvard and Massachusetts Institute of Technology (MIT), Cambridge, United States of America; 3 Section for Immunogenetics and Transplantation Immunology of the Department of Immunohematology and Blood Transfusion (IHB), Leiden University Medical Center (LUMC), Leiden, The Netherlands; 4 Genetics Department, University Medical Centre Groningen and University of Groningen, Groningen, The Netherlands; 5 Department of Pediatrics and European Laboratory for the Investigation of Food-Induced Diseases, University Federico II Naples, Napels, Italy; 6 Pediatric Gastroenterology Unit, Fundación Investigación Hospital La Fe, Valencia, Spain; 7 Institute of Cell and Molecular Science, Barts and the London School of Medicine and Dentistry, London, United Kingdom; 8 Department of Pathology, Vrije Universiteit (VU) University Medical Centre, Amsterdam, The Netherlands; 9 Division of Genetics, Brigham and Women's Hospital, Harvard-Partners Center for Genetics and Genomics, Boston, United States of America; Vrije Universiteit Medical Centre, Netherlands

## Abstract

**Background:**

The HLA genes, located in the MHC region on chromosome 6p21.3, play an important role in many autoimmune disorders, such as celiac disease (CD), type 1 diabetes (T1D), rheumatoid arthritis, multiple sclerosis, psoriasis and others. Known HLA variants that confer risk to CD, for example, include DQA1*05/DQB1*02 (DQ2.5) and DQA1*03/DQB1*0302 (DQ8). To diagnose the majority of CD patients and to study disease susceptibility and progression, typing these strongly associated HLA risk factors is of utmost importance. However, current genotyping methods for HLA risk factors involve many reactions, and are complicated and expensive. We sought a simple experimental approach using tagging SNPs that predict the CD-associated HLA risk factors.

**Methodology:**

Our tagging approach exploits linkage disequilibrium between single nucleotide polymorphism (SNPs) and the CD-associated HLA risk factors DQ2.5 and DQ8 that indicate direct risk, and DQA1*0201/DQB1*0202 (DQ2.2) and DQA1*0505/DQB1*0301 (DQ7) that attribute to the risk of DQ2.5 to CD. To evaluate the predictive power of this approach, we performed an empirical comparison of the predicted DQ types, based on these six tag SNPs, with those executed with current validated laboratory typing methods of the *HLA-DQA1* and -*DQB1* genes in three large cohorts. The results were validated in three European celiac populations.

**Conclusion:**

Using this method, only six SNPs were needed to predict the risk types carried by >95% of CD patients. We determined that for this tagging approach the sensitivity was >0.991, specificity >0.996 and the predictive value >0.948. Our results show that this tag SNP method is very accurate and provides an excellent basis for population screening for CD. This method is broadly applicable in European populations.

## Introduction

The HLA genes, located in the major histocompatibility (MHC) region on chromosome 6p21.3, play a role in multiple autoimmune disorders, like celiac disease (CD), type 1 diabetes (T1D), rheumatoid arthritis, multiple sclerosis, psoriasis and others [1]–[3]. The MHC region is highly polymorphic and some genes in this region are involved in multiple disorders. For example, the *HLA-DQA1* and *-DQB1* genes have alleles that confer risk to both CD and T1D. In most autoimmune diseases not all patients carry the same risk alleles, and multiple risk alleles are likely to be involved [2].

**Figure 1:**
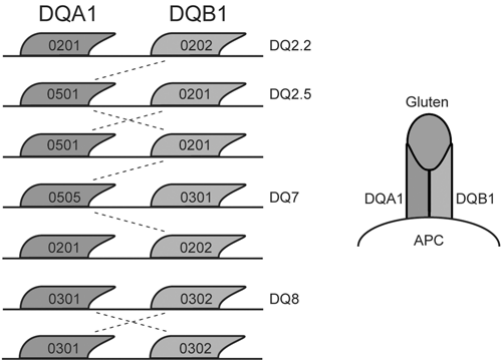
HLA-DQA1* and -DQB1* together form heterodimers of which DQ2.5 and DQ8, either in homozygous or heterozygous state, confer risk to CD due to their ability to present gluten to T cells. DQ2.2 and DQ7 can only confer risk to CD when both are present together or with DQ2.5 (trans effect, see dashed lines). See Table 1 for the possible combinations, the number of risk molecules and the associated risk.

CD, the most common intolerance to a dietary component in Western society, is sustained by an abnormal T cell response to gluten as an environmental factor and is strongly associated with HLA class II genes. Almost 95% of CD patients carry at least one of the two risk molecules DQA1*05/DQB1*02 (i.e. haplotype DQ2.5) and DQA1*03/DQB1*0302 (i.e. haplotype DQ8) [2], [4]–[7]. The molecules encoded by the CD-associated *HLA-DQA1* and -*DQB1* genes form DQα and DQβ heterodimers, which can lead to several functional molecules of which one to four copies can be made. A few variants of these genes predispose to CD (either alone or in combination) when gluten peptides, present in wheat, barley and rye, are presented to CD4+ cells in the lamina propria [8], [9]. The most important risk factor for CD is the DQ2.5 haplotype (see Figure 1 and Table 1) [5], [10], [11], with the highest risk in individuals homozygous for this haplotype [8], [12], or those who have a single copy of DQ2.5 and one copy of DQA1*0201/DQB1*0202 (i.e. haplotype DQ2.2) molecules, haplotype DQ8, or DQA1*0505/DQB1*0301 (i.e. haplotype DQ7). The frequency of these alleles in the general population is substantial (>25%), suggesting that these variants are necessary for disease development but not sufficient.

**Table 1:** Genetic risk associated with the different HLA-DQ molecules

DQ molecule 1	DQ molecule 2	Number of functional copies	Genetic risk
DQ2.5	Non-CD risk types	≥1	5.5
DQ2.5	DQ2.5	4	13.1
DQ2.5	no DQ2.2, DQ2.5, DQ7	1	1.3
DQ2.5	no DQ2.5	1–2	2.5
DQ2.5	DQ2.2	2	10.1
DQ2.2 or DQ2.5	Non-CD risk types	1–4	24.4
DQ2.2	DQ7	1	1.8*
DQ2.2	no DQ2.5, DQ7	0	-
DQ7	no DQ2.2, DQ2.5	0	-
DQ2.5	DQ7	2	-
DQ8	Non-CD risk types	1	-
DQ8	DQ8	4	-

**b)** Combinations of the DQ molecules on the two chromosomes, the number of functional copies and the genetic risk associated to coeliac disease (calculated using the CD cohort and the blood bank control cohort).

* This risk increases to 4.1 in the DQ2.5 negative group.

Family-based or population-based screening for the CD risk variants has important diagnostic value in supporting the diagnosis of CD when these alleles are present, and the possibility of CD is minimized when they are not present (they have a high negative predictive value). In a recent study by Bourgey et al. [13] it was shown that the risk estimates of a sibling of a CD patient ranges from 0.1% to 29% when HLA-DQ information is included, compared to the overall risk for sibs of approx. 10%. CD affects almost 1% of the population, although it is estimated that most cases remain undiagnosed [14]. Since untreated CD can cause long-term health problems, targeted screening in e.g. families for CD could identify such undiagnosed individuals and prevent life-long symptoms and complications.

Testing for HLA risk molecules is routinely performed using specialized kits, but they often require 24–60 reactions, multiple steps, like amplification and hybridization to a membrane, special software or expertise in analyzing the results and most of these methods are expensive (e.g. DNA PCR-single-strand conformation polymorphism (PCR-SSCP) [15], PCR and sequence specific oligonucleotide probing (PCR-SSOP) [16], PCR-sequence specific primer kits (PCR-SSP [17], PCR-reverse line blot (PCR-RLB) [18]). Direct typing of the genetic variants that encode the HLA alleles is usually very difficult since most of these variants are surrounded by too many other variants that interfere with primer annealing.

The International HapMap Project and an independent MHC-focused effort [19], [20] have empirically determined the fine-scale patterns of linkage disequilibrium (LD) among local sequence polymorphisms in four population samples. With these resources it is now possible to pick tag SNPs that are in LD with specific HLA variants of interest (i.e. have high r^2^ values). Recently, an LD-based tagging approach was shown to predict HLA-DQ2.2 and –DQ2.5 alleles in independent patient samples with a high degree of accuracy [20].

In this study we selected tag SNPs to predict DQ2.2, DQ2.5, DQ7 and DQ8, in three cohorts: CD patients, non-CD trio control families, and blood bank controls (HLA typing was available for all individuals). We then examined the sensitivity, specificity, predictive value and the correlation between the SNP-based test and the true HLA variant (r^2^). This study represents a first step towards providing a cost-effective population screening method for CD.

## Results

A total of six SNPs were needed to predict the DQ2.2, DQ2.5, DQ7 and DQ8 risk types for CD. Typing was done in three different cohorts comprising a total of 754 persons (1512 alleles). Dropout rates for these SNPs were <3.3% as described in Table S1. All SNPs were in Hardy-Weinberg equilibrium and no Mendelian errors were observed in the trios.

We observed a high correlation between the three cohorts for the sensitivity, specificity, PPV and the r^2^. We grouped the results of the three different cohorts (Table 2) and show the results of each separate group in Table S2. Specifications of the individuals of whom the predicted HLA-DQ typing results did not correspond with those from the typing centres are shown in Table S3. For all false-positive and false-negative typings, the SNPs were retyped and all official typings were either double checked or retyped. However it was not always possible to use DNA from one tube for both the SNP retyping and official retyping, so we cannot fully rule out the possibility of DNA switching leading to false-positive or false-negative results.

**Table 2:** Prediction results for combined cohorts

		DQ2.2				
		+	−	total	sensitivity	0.992
SNP prediction	+	126	3	129	specificity	0.998
	−	1	1318	1319	positive predictive value	0.977
	total	127	1321	1448	r-squared	0.966
		DQ2.5				
		+	−	total	sensitivity	1.000
SNP prediction	+	569	1	570	specificity	0.999
	−	0	888	888	positive predictive value	0.998
	total	569	889	1458	r-squared	0.997
		DQ7				
		+	−	total	sensitivity	1.000
SNP prediction	+	94	4	98	specificity	0.997
	−	0	1372	1372	positive predictive value	0.959
	total	94	1376	1470	r-squared	0.956
						0.069
		DQ8				
		+	−	total	sensitivity	0.991
SNP prediction	+	110	6	116	specificity	0.996
	−	1	1367	1368	positive predictive value	0.948
	total	111	1373	1484	r-squared	0.935

For each DQ type we used all persons with non-missing data for the relevant SNPs. A person with missing data for DQ2.5, for example, was excluded from the DQ2.5 analysis, but could be used for the other analyses if genotypes relevant for the other DQ types were present.

At first the sensitivity and specificity for DQ2.2 was high and accurate but the predictive value was low. The SNPs for DQ2.2 (rs2395182, rs7775228) not only tagged DQ2.2 but also included the relatively infrequent DQ4 allele. We therefore decided to tag DQ4 as well (rs4713586) making it possible to call a person DQ2.2 when the alleles were positive for DQ2.2 and negative for DQ4. This led to three tag SNPs being needed for the prediction of DQ2.2, with an overall sensitivity of 0.992, a specificity of 0.998 and a PPV of 0.977. Only four of the 1448 tested chromosomes gave false results (0.28%).

The tag SNP selected for prediction of DQ2.5 (rs2187668) showed an overall sensitivity of 1.000, a specificity of 0.999 and a PPV of 0.998. Only one of the 1458 tested chromosomes gave false results (0.07%). This person did indeed carry half of the DQ2.5 haplotype (DQA1*0501) (see Table S3b, person no. 10).

The tag SNP for DQ7 (rs4639334) showed an overall sensitivity of 1.000, a specificity of 0.997 and a PPV of 0.959. Four of the 1470 tested chromosomes gave false results (0.27%), of which three carried a rare haplotype consisting of half of the DQ7 haplotype. Two of these three carried a combination of the DQA1*0505 allele of DQ7 with the DQB1*0302 of DQ8 (see results of DQ8 as well) and were predicted to be both DQ7 and DQ8 (see Table S3b, person nos. 11 and 12). For one of these persons we typed the parents and we could see a transmission of this combined haplotype in the family.

The tag SNP for DQ8 (rs7454108) showed an overall sensitivity of 0.991, a specificity of 0.996 and a PPV of 0.948. Seven of the 1484 tested chromosomes gave false results (0.5%).

Accepting the prediction of these half haplotypes as good predictions of the risk alleles increases the sensitivity, specificity and PPV slightly.

To validate our results in other populations, we additionally tested the same SNP panel in 76 HLA-typed individuals from the “preventCD” study populations (a family based celiac disease study) from Valencia (Spain) (n = 32) and Naples (Italy) (n = 44). In addition, we previously reported on the performance of the DQ2.5 predictive SNP rs2187668 SNP in 262 HLA-typed celiac cases from the UK as part of a genome-wide association study [23]. The overall sensitivity and specificity in the UK celiac population was similar to what was observed in the Dutch celiac population. In the Spanish and Italian celiac cohort a few samples showed discordance for the DQ2.5 and DQ7 haplotype, giving rise to slightly lower r^2^ values for this prediction. Prediction values for DQ2.2 and DQ8 in both Spanish and Italian celiac samples were similar to the Dutch results (Table S4).

## Discussion

In this study we used a tag SNP approach to predict whether an individual carried the risk DQ types (formed by variants in the *HLA-DQA1* and -*DQB1* genes) that are positively associated with CD. Using this method, only six SNPs were needed to predict the DQ2.2, DQ2.5, DQ7 and DQ8 risk types carried by >95% of CD patients. We determined that for this tagging approach the sensitivity was >0.991, specificity >0.996 and the predictive value >0.948.

Most of the patients without DQ2.5 and DQ8, carried half of the DQ2.5 or DQ2.2 molecule (either HLA-DQA1*05 or -DQB1*0202) suggesting that carrying part of the risk molecules has functional implications for the risk of CD [4], [22]. Of our patient group 98.4% carry one of the risk groups (DQ2.2, DQ2.5, DQ7, DQ8 or the DQ types that have half of the risk haplotypes) and 98.3% of all patients were correctly predicted using our method. Overall, the sensitivity was 0.997, the specificity was >0.929 and predictive value was >0.987 when taking into account that some of the false predictions included an allele that is part of a risk haplotype (e.g. the HLA-DQA1*05 allele which is part of the DQ2.5 haplotype, see person nos. 10–12 in Table S3b).

This method also allowed us to determine whether an individual was homozygous or heterozygous for the risk molecule. Vader et al. demonstrated a >4-fold higher T cell response when gluten was presented by antigen-presenting cells from DQ2 homozygous patients compared to DQ2 heterozygous patients, thereby providing an explanation for the dose-effect of risk molecules for developing CD [9]. Al-Toma et al. showed that homozygosity for DQ2.5 was seen more than twice as often in individuals that developed refractory celiac disease and enteropathy-associated T-cell lymphoma, associated with a high morbidity, than in uncomplicated CD [12].

Reinton et al. developed a real-time PCR method for detecting CD-associated HLA risk alleles [23]. This method requires 11 reactions and even more if homozygous persons for the HLA-risk alleles need to be distinguished from heterozygous persons. It is not clear whether this real-time PCR method can be easily applied to high-throughput typing or not, whereas our method can. We can perform PCR reactions in multiple PCR machines at the same time and use the ABI PRISM 7900 HT system only for end-point measurements. Moreover, Reinton et al. only used a relatively small set of samples to test their method, making it difficult to determine its robustness.

De Bakker et al. showed two examples that used the tagging method for CD and systemic lupus erythematosus [20]. They chose two SNPs to capture DQ2.2 and DQ2.5 in the same CD cohort (N = 330) that we have used in this paper. The rs4988889(T), rs2858331(C) haplotype was used to determine the presence of DQ2.2 and the rs4988889(T), rs2858331(T) haplotype was used to determine DQ2.5. Although the SNPs look promising in determining DQ2.5 homozygosity or DQ2.2/DQ2.5 heterozygosity, it was often difficult to distinguish DQ2.2/X heterozygous from the DQ2.5/X heterozygous individuals (X is any other allele excluding DQ2.2 or DQ2.5), due to phase uncertainty of the alleles at the two SNPs. An individual who is heterozygous for rs4988889 (G/T) has one copy of DQ2.2 or DQ2.5. If he/she is also heterozygous for rs2858331 (C/T), then it is uncertain which of these alleles (either C or T) is on the same chromosome as the T allele of rs498889, and therefore forms either DQ2.2 or DQ2.5. In contrast to these examples are the SNPs we used in the current study, which are capable of determining whether an individual is homozygous for DQ2.2 or DQ2.5, heterozygous for DQ2.2 or DQ2.5, or does not possess the DQ2.2 or DQ2.5 haplotype at all.

We expect the chosen tag SNPs to be transferable within European populations given the strong conservation of the HLA-DR3-DQ2 haplotype. De Bakker et al. has given two examples that show that tag SNPs chosen from the CEU panel (CEPH (Utah residents with ancestry from northern and western Europe)) are applicable in other populations and our data also gives similar r^2^ values to the CEU panel [20]. The r^2^ might be somewhat higher or lower in the population that it is applied to, with a resulting gain or loss of power, but the differences observed are minimal. We have also seen that the tag SNP for DQ2.5 tested in an UK population gives comparable results (UK population: r^2^ 0.96, Dutch population: r^2^ 0.99) [21]. Analysing the tag SNPs in two southern European populations – from Italy and Spain – gives lower r^2^ values compared to the Dutch, especially for the DQ2.5 and DQ7 genotypes. Overall, however, both populations show high sensitivity and specificity. The lower r^2^ values in the Spanish and Italian populations could be caused by the presence of rare southern Europe-specific haplotypes, or due to inaccuracies of the original HLA II DQ2 and DQ8 haplotype identification by an SSP-based method. The few false-negative or false-positive predictions observed in the Dutch celiac population may also be explained by the presence of rare haplotypes. The transferability of this method in populations that have known differences in LD structure, like African, Japanese, Chinese, still needs to be determined. The six SNPs should be tested in these populations and, using the data of de Bakker et al., other tag SNPs might be found that perform equally or perhaps better than the six SNPs presented in this paper [20].

Our method is attractive because it is cost-effective and the experimental procedures are straightforward, using routine genotyping equipment. Although more work is needed to validate this approach for diagnostic purposes, our work provides a foundation for simple SNP-based population screening for CD. Population screening has been discussed for a long time and it would certainly be helpful in finding all the undiagnosed CD patients, which could prevent negative outcomes [14]. However no steps have actually been taken towards implementing it largely due to the cost of classical HLA typing and the need to repeat serology tests during an individual's lifetime. Since an individual can develop antibodies to CD later in life, repeated testing would impose an extra burden on people who might not be at any risk. It would be easy, cheap and quick to use our tag SNP method to determine which part of the population (∼25%) needs to be screened more extensively for CD. As this test requires very little DNA and is fairly insensitive to DNA quality, it can also be used with DNA material isolated from e.g. biopsies, whole-genome amplified DNA, and DNA isolated from FTA cards. Furthermore, this test determines which individuals are not at risk for developing CD and who therefore do not need further serology tests.

We have described a robust method to predict the risk DQ types involved in CD with high accuracy. This method can also be applied to T1D, in which DQ2.5 and DQ8 are also known risk factors, or more generally for other immune-related diseases with known HLA risk alleles.

## Materials and Methods

### DNA samples

DNA was available from three different cohorts; these were used to study different aspects of the tag SNP method. The CD cohort had a high number of individuals with HLA-DQ2 risk variants, which was useful for testing the positive predictive value. The trio control cohort enabled us to check for Mendelian errors (which were not observed). while the blood bank controls gave a better view of the robustness of the method in the general population. The first cohort consisted of 330 unrelated CD patients of Dutch Caucasian origin [24]. Only CD patients diagnosed according to revised ESPGHAN criteria and with a Marsh III lesion confirmed by duodenal biopsy sampling were selected for this study, as described by Van Belzen et al. [25] and Walker-Smith et al. [26]. A cohort of population-based control trios was derived from families without a history of CD [27]. The 86 control trios were selected for the presence of at least one parent carrying haplotype DQ2.5 and were all of Dutch Caucasian origin. HLA typing data was available for 207 of the 264 persons in the 86 trios (see below), of these 2 persons dropped out for all SNPs typed and were excluded from this paper. The blood bank cohort was part of the ITI two panel (the ITI panel is a DNA panel from the Immunogenetics and Transplantation Immunology Section of the LUMC) and consisted of 219 unrelated, randomly selected, Dutch blood donors. We studied a total of 754 persons. The study was approved by the Medical Ethics Committee of the University Medical Centre Utrecht, and informed consent was obtained from the participants.

The replication cohort includes 32 HLA-typed Spanish celiac samples from Valencia and 44 HLA-typed Italian celiac samples from Naples. These two study samples form part of the “preventCD” study, a European multicenter study. The 262 HLA-typed UK celiac cases were recently included in a genome-wide association study [21].

### HLA typing

The CD cohort and the trio control cohort were typed for *HLA-DQA1* and -*DQB1* genes using a classical PCR-SSCP/heteroduplex method in an official HLA typing laboratory as described elsewhere [12], [15]. Full HLA-DQA1 and -DQB1 typing was available for the entire CD cohort. For the trio control cohort, full HLA-DQA1 and -DQB1 typing was available for the child and both parents in 34 trios and for the child and one of the parents in 51 trios, and for one trio only one person could be SNP typed leading to a total of 205 persons available for analyses. For the blood bank control cohort, full (four digit) HLA-DRB1, -DQA1 and -DQB1 typing was performed by PCR-SSCP using locally produced and slightly modified primer mixes [28]. The typing of this cohort was performed in the European Foundation of Immunogenetics (EFI)-accredited HLA laboratory of the Department of IHB, LUMC, Leiden. The typing of UK samples were performed at the Transplant Immunology laboratory, Oxford Radcliffe Hospitals NHS Trust using the PCR-SSCP method. The Italian and Spanish samples were HLA typed by Eurospital (Italy) using the Eu-DQ kit.

### Tag SNP selection

Tag SNPs were selected that captured the following HLA types: DQ2.2 (2 SNPs for DQ2.2 and one SNP to exclude DQ4 from the DQ2.2 group), DQ2.5 (1 SNP), DQ7 (1 SNP), and DQ8 (1 SNP) (see Table 3). DQ2.5 and DQ8 are risk factors for CD and are carried by ∼95% of CD patients [4], [22]. The HLA-DQA1*0505 allele of DQ7 and HLA-DQA1*0501 allele of DQ2.5 only differ by one or a few base pairs and are thought to have the same functional properties. This also holds for the HLA-DQB1*0202 allele of DQ2.2 and the HLA-DQB1*0201 allele of DQ2.5. Most of the CD patients who do not carry DQ2.5 or DQ8, carry half of the DQ2.5 or DQ2.2 molecule (that is either HLA-DQA1*05 or -DQB1*0202) suggesting that carrying part of the risk molecules has functional implications for the risk of CD [4].

**Table 3:** DQ molecules and tested tag SNPs

DQ type	DQA1	DQB1	DR	tag SNP	Positive predicting allele(s) (freqCEU)	tag SNP	Negative predicting allele
DQ2.2	0201	0202	7	rs2395182, rs7775228	T (0.71), G (0.10)	rs4713586	G (0.025)
DQ2.5	0501	0201	3	rs2187668	T (0.09)		
DQ7	0505	0301	5	rs4639334	A (0.09)		
DQ8	0301	0302	4	rs7454108	G (0.18)		

**a)** DQ molecules, the corresponding HLA-DQA1^*^ and -DQB1^*^ alleles, with the DR type and the tag SNPs. A person that has the T,G,A haplotype for rs2395182, rs7775228, rs4713586, is a DQ2.2.

A person that has the T,G,G haplotype for rs2395182, rs7775228, rs4713586, is not a DQ2.2 but a DQ4.

Freq(CEU) – frequency of annotated alleles in CEU HapMap population

Tag SNP selection was based on genotype data collected in the classical HLA genes and >7,500 common SNP and deletion-insertion polymorphisms across the extended human MHC region [20]. We used Tagger [29] to derive SNP-based tests to capture each DQ type in the extended CEU analysis panel (Utah residents with northern and western European ancestry). We first found the SNPs that have the highest r^2^ to a DQ type. We proceeded with multi-SNP (haplotype) tests to achieve higher r^2^ with which a DQ type was captured (if r^2^ <1). For DQ2.2, multiple SNPs were needed that in combination capture this HLA allele. Since there is a lot of variation in the MHC region that can interfere in primer annealing, we only selected SNPs that could be typed using conventional methods (e.g. Taqman).

### Tag SNP typing

The tag SNPs in all samples except for the UK cohort were obtained as Assay on Demand (rs2395182, rs4713586, rs4639334) or Assay by Design (rs7454108, rs7775228, rs2187668) from Applied Biosystems (Applied Biosystems, Foster City, California, USA) (see Table S1 for assay numbers or primer sequences and their allele labeling). Samples were genotyped using the manufacturer's instructions and analyzed on an ABI PRISM 7900 HT system (Applied Biosystems). All SNPs were typed using the standard amplification protocol as supplied by Applied Biosystems. We obtained end-point measurements for the analysis. Dropout rates were below 3.57% and are shown in Table S1 for each individual SNP. No Mendelian errors were observed for the SNPs in the trio control cohort. Genotyping of rs2187668 SNP in UK samples was obtained as part of a genome-wide association study using the Illumina 300K chip [21].

### Analyses

The HLA-DQA1 and -DQB1 genotypes as determined at the HLA-typing centres were used to establish the corresponding DQ types (see Figure 1). Due to the high linkage disequilibrium in the MHC region, only a limited set of DQA1*-DQB1* haplotypes (DQ types) are observed in the general population (see http://depts.washington.edu/rhwlab/resMat/dq/linkage.html for an example of common combinations of DQA1* and DQB1* alleles in the Caucasian population), resulting in only a few instances that did not correspond to canonical DQ types. For the prediction method we inferred DQ types from the tag SNPs. DQ types were determined according to the predicting alleles (see Table 3, e.g. a person was called homozygous DQ8 if rs7454108 was homozygous G, or heterozygous DQ8 if rs7454108 was heterozygous G/A). Only individuals with non-missing data were used for comparing the official typing and the prediction method. DQ types based on the official typing and those from the tag SNP typing method were compared to examine the sensitivity, specificity, positive predictive value (PPV) and r^2^.

## Supporting Information

Table S1 (0.05 MB DOC)

Table S2 (0.13 MB DOC)

Table S3 (0.05 MB DOC)

Table S4 (0.08 MB DOC)
