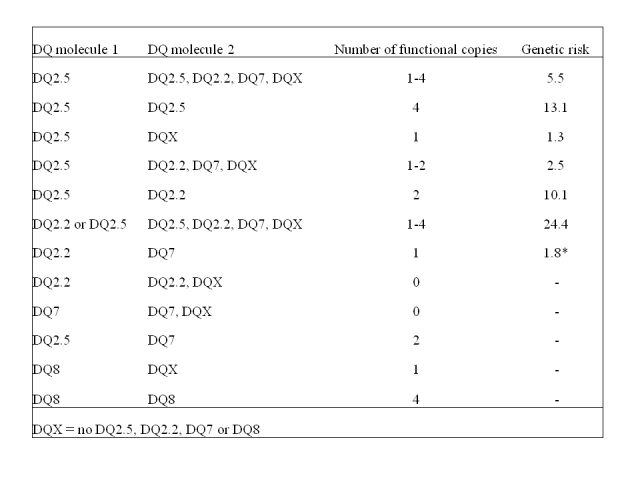# Correction: Effective Detection of Human Leukocyte Antigen Risk Alleles in Celiac Disease Using Tag Single Nucleotide Polymorphisms

**DOI:** 10.1371/annotation/53480f56-4ef7-4877-ace7-e5892d392cce

**Published:** 2009-05-15

**Authors:** Alienke J. Monsuur, Paul I. W. de Bakker, Alexandra Zhernakova, Dalila Pinto, Willem Verduijn, Jihane Romanos, Renata Auricchio, Ana Lopez, David A. van Heel, J. Bart A Crusius, Cisca Wijmenga

Table 1 contains typos. Please view the corrected Table 1 here: 

**Figure:**