# Supplementary material for: Effective Detection of Human Leukocyte Antigen Risk Alleles in Celiac Disease Using Tag Single Nucleotide Polymorphisms
Source: PLoS One. 2008 May 28;3(5):e2270. doi: 10.1371/journal.pone.0002270 (PMC2386975; doi:10.1371/journal.pone.0002270)
Supplement: Table S1 — (0.05 MB DOC) [file pone.0002270.s001.doc]

**Table S1.** Information on the tested tag SNPs: Alleles and allele calling, assays, primer sequences, drop-out rates and location in chromosome 6p21.3 region.

| rs number | Allele call | | Assay type | Assay number | Basepair location | Drop-out rate |
| --- | --- | --- | --- | --- | --- | --- |
|  | VIC | FAM |  |  |  | % |
| rs2395182 | G | T | On Demand | C__11409965_10 | 32521295 | 1.1 |
| rs4713586 | A | G | On Demand | C__27950246_10 | 32767560 | 2.8 |
| rs4639334 | A | G | On Demand | C__42975350_10 | 32710192 | 2.5 |
| rs7454108 | G | A | By Design |  | 32789461 | 1.6 |
| rs7775228 | A | G | By Design |  | 32766057 | 0.7 |
| rs2187668 | T | C | By Design |  | 32713862 | 3.3 |

| rs number | Primer sequence | | Reporter sequence | |
| --- | --- | --- | --- | --- |
|  | Forward | Reverse | VIC | FAM |
| rs7454108 | ACTATTATTTCTCCAAGTTCTGACTTCCCT | GCCAAGTTGGAATAAGCCCACTATA | CAAAATAGCATGAGTATTAG | AAAATAGCATGAATATTAG |
| rs7775228 | AGGAAAGGAACTATCTGGGTATGGA | TGCAAAGCCCCTTTATCATTATCCT | TTCAATCACAATCTTGC | TCAATCACAGTCTTGC |
| rs2187668 | GTGAGGTGACACATATGAGGCAG | GGCTGAATGCCTTCAACAATCATTT | CTGAGAGTAAATGAGGACC | TGAGAGTAAGTGAGGACC |
