# Supplementary material for: Effective Detection of Human Leukocyte Antigen Risk Alleles in Celiac Disease Using Tag Single Nucleotide Polymorphisms
Source: PLoS One. 2008 May 28;3(5):e2270. doi: 10.1371/journal.pone.0002270 (PMC2386975; doi:10.1371/journal.pone.0002270)
Supplement: Table S2 — (0.13 MB DOC) [file pone.0002270.s002.doc]

**Table S2.** Predictive results. a) DQ2.2 per cohort, b) DQ2.5 per cohort, c) DQ7 per cohort, d) DQ8 per cohort.

a

| Blood bank cohort | | DQ2.2 | |  |  |  |
| --- | --- | --- | --- | --- | --- | --- |
|  |  | + | - |  | sensitivity | 1,000 |
| SNP prediction | + | 34 | 2 | 36 | specificity | 0,995 |
|  | - | 0 | 382 | 382 | positive predictive value | 0,944 |
|  |  | 34 | 384 | 418 | r-squared | 0,939 |
|  |  |  |  |  |  |  |
| Cases |  | DQ2.2 | |  |  |  |
|  |  | + | - |  | sensitivity | 0,987 |
| SNP prediction | + | 77 | 1 | 78 | specificity | 0,998 |
|  | - | 1 | 559 | 560 | positive predictive value | 0,987 |
|  |  | 78 | 560 | 638 | r-squared | 0,971 |
|  |  |  |  |  |  |  |
| Control trios | | DQ2.2 | |  |  |  |
|  |  | + | - |  | sensitivity | 1,000 |
| SNP prediction | + | 15 | 0 | 15 | specificity | 1,000 |
|  | - | 0 | 377 | 377 | positive predictive value | 1,000 |
|  |  | 15 | 377 | 392 | r-squared | 1,000 |

b

| Blood bank cohort | | DQ2.5 | |  |  |  |
| --- | --- | --- | --- | --- | --- | --- |
|  |  | + | - |  | sensitivity | 1,000 |
| SNP prediction | + | 57 | 0 | 57 | specificity | 1,000 |
|  | - | 0 | 369 | 369 | positive predictive value | 1,000 |
|  |  | 57 | 369 | 426 | r-squared | 1,000 |
|  |  |  |  |  |  |  |
| Cases |  | DQ2.5 | |  |  |  |
|  |  | + | - |  | sensitivity | 1,000 |
| SNP prediction | + | 354 | 1 | 355 | specificity | 0,996 |
|  | - | 0 | 281 | 281 | positive predictive value | 0,997 |
|  |  | 354 | 282 | 636 | r-squared | 0,994 |
|  |  |  |  |  |  |  |
| Control trios | | DQ2.5 | |  |  |  |
|  |  | + | - |  | sensitivity | 1,000 |
| SNP prediction | + | 158 | 0 | 158 | specificity | 1,000 |
|  | - | 0 | 238 | 238 | positive predictive value | 1,000 |
|  |  | 158 | 238 | 396 | r-squared | 1,000 |

c

| Blood bank cohort | | DQ7 | |  |  |  |
| --- | --- | --- | --- | --- | --- | --- |
|  |  | + | - |  | sensitivity | 1,000 |
| SNP prediction | + | 35 | 1 | 36 | specificity | 0,997 |
|  | - | 0 | 384 | 384 | positive predictive value | 0,972 |
|  |  | 35 | 385 | 420 | r-squared | 0,970 |
|  |  |  |  |  |  |  |
| Cases |  | DQ7 | |  |  |  |
|  |  | + | - |  | sensitivity | 1,000 |
| SNP prediction | + | 29 | 2 | 31 | specificity | 0,997 |
|  | - | 0 | 613 | 613 | positive predictive value | 0,935 |
|  |  | 29 | 615 | 644 | r-squared | 0,932 |
|  |  |  |  |  |  |  |
| Control trios | | DQ7 | |  |  |  |
|  |  | + | - |  | sensitivity | 1,000 |
| SNP prediction | + | 30 | 1 | 31 | specificity | 0,997 |
|  | - | 0 | 375 | 375 | positive predictive value | 0,968 |
|  |  | 30 | 376 | 406 | r-squared | 0,965 |

d

| Blood bank cohort | | DQ8 | |  |  |  |
| --- | --- | --- | --- | --- | --- | --- |
|  |  | + | - |  | sensitivity | 1,000 |
| SNP prediction | + | 43 | 1 | 44 | specificity | 0,997 |
|  | - | 0 | 388 | 388 | positive predictive value | 0,977 |
|  |  | 43 | 389 | 432 | r-squared | 0,975 |
|  |  |  |  |  |  |  |
| Cases |  | DQ8 | |  |  |  |
|  |  | + | - |  | sensitivity | 0,973 |
| SNP prediction | + | 36 | 3 | 39 | specificity | 0,995 |
|  | - | 1 | 610 | 611 | positive predictive value | 0,923 |
|  |  | 37 | 613 | 650 | r-squared | 0,892 |
|  |  |  |  |  |  |  |
| Control trios | | DQ8 | |  |  |  |
|  |  | + | - |  | sensitivity | 1,000 |
| SNP prediction | + | 31 | 2 | 33 | specificity | 0,995 |
|  | - | 0 | 369 | 369 | positive predictive value | 0,939 |
|  |  | 31 | 371 | 402 | r-squared | 0,934 |
