# Supplementary material for: Effective Detection of Human Leukocyte Antigen Risk Alleles in Celiac Disease Using Tag Single Nucleotide Polymorphisms
Source: PLoS One. 2008 May 28;3(5):e2270. doi: 10.1371/journal.pone.0002270 (PMC2386975; doi:10.1371/journal.pone.0002270)
Supplement: Table S3 — (0.05 MB DOC) [file pone.0002270.s003.doc]

**Table S3.** False-positive and false-negative results. DQ types, predicted using the tag SNPs are shown as well as the official HLA-DQA1 and -DQB1 typing of both chromosomes, with the corresponding DQ.

a) Wrongly predicted DQ types; b) wrongly predicted results from individuals who carry part of the risk haplotypes for CD (in rare DQA1*/DQB1*combinations) and who therefore contributed to the CD risk. DQX are rare combinations for which there is no generally used DQ name.

a

| Cohort | Prediction of DQ | | | Chromosome 1* | | | Chromosome 2* | | | Remark |
| --- | --- | --- | --- | --- | --- | --- | --- | --- | --- | --- |
|  | Allele 1 | Allele 2 | Allele 3 | DQA1 | DQB1 | DQ | DQA1 | DQB1 | DQ |  |
| 1. Blood bank control | DQ2.2 | DQ7 |  | 0103 | 0601 | DQ6 | 0505 | 0301 | DQ7 | DQ7 correctly predicted,  DQ2.2 predicted but not present |
| 2. Blood bank control | DQ2.2 | - |  | 0103 | 0601 | DQ6 | 0201 | 0303 | DQ9 | DQ2.2 predicted but not present |
| 3. CD case | DQ2.2 | DQ7 |  | 0102 | 0602/03 | DQ6 | 03 | 0302 | DQ8 | DQ2.2 and DQ7 predicted but not present,  DQ8 present but not predicted |
| 4. CD case | DQ2.5 |  |  | 0201 | 02 | DQ2.2 | 0501 | 02 | DQ2.5 | DQ2.5 is predicted and present,  DQ2.2 is present but not predicted |
| 5. Blood bank control | DQ7 | DQ7 |  | 0104 | 0503 | DQ5 | 0505 | 0301 | DQ7 | Homozygosity for DQ7 is predicted,  but the person is heterozygous for DQ7 |
| 6. Blood bank control | DQ7 | DQ8 |  | 0103 | 0603 | DQ6 | 0505 | 0301 | DQ7 | DQ7 is predicted and present,  DQ8 is predicted but not present |
| 7. CD case | DQ2.5 | DQ2.5 | DQ8 | 0501 | 02 | DQ2.5 | 0501 | 02 | DQ2.5 | Homozygosity for DQ2.5 is correctly predicted,  DQ8 is also predicted but not present |
| 8. CD case | DQ2.5 | DQ8 |  | 0101/02 | 0602/03 | DQ6 | 0501 | 02 | DQ2.5 | DQ2.5 is predicted and present, DQ8 is predicted but not present |
| 9. Trio control | DQ2.5 | DQ8 |  | 01 | 0501 | DQ5 | 0501 | 02 | DQ2.5 | DQ2.5 is predicted and present,  DQ8 is predicted but not present |

b

| Cohort | Prediction of DQ | | | Chromosome 1 | | | Chromosome 2 | | | Remark |
| --- | --- | --- | --- | --- | --- | --- | --- | --- | --- | --- |
|  | Allele 1 | Allele 2 | Allele 3 | DQA1 | DQB1 | DQ | DQA1 | DQB1 | DQ |  |
| 10. CD case | DQ2.5 | DQ2.5 |  | 0501 | 0602/03 | DQX | 0501 | 02 | DQ2.5 | Homozygosity for DQ2.5 is predicted,  but the person carries one complete DQ2.5  and one half DQ2.5 (the DAQ1*0501 allele of DQX), this is a rare combination |
| 11. Trio control | DQ2.5 | DQ7 | DQ8 | 0501 | 0302 | DQX | 0501 | 02 | DQ2.5 | DQ2.5 is predicted and present, DQ7 and 8 are also predicted, but they are present in  a rare combination (DQA1*0505 from DQ7 and DQB1*0302 from DQ8), this rare combination is transmitted in the trio family |
| 12. CD case | DQ2.5 | DQ7 | DQ8 | 0501 | 0302 | DQX | 0501 | 02 | DQ2.5 | DQ2.5 is predicted and present, DQ7 and 8 are also predicted, but they are present in a rare combination (DQA1*0505 from DQ7 and DQB1*0302 from DQ8) |
