# Supplementary material for: Effective Detection of Human Leukocyte Antigen Risk Alleles in Celiac Disease Using Tag Single Nucleotide Polymorphisms
Source: PLoS One. 2008 May 28;3(5):e2270. doi: 10.1371/journal.pone.0002270 (PMC2386975; doi:10.1371/journal.pone.0002270)
Supplement: Table S4 — (0.08 MB DOC) [file pone.0002270.s004.doc]

**Table S4.** Validation of tagging SNPs in non-Dutch populations: Predictive results for all SNPs in Spanish (n=32) and Italian (n=44) cohort and for DQ2.5 tag SNP in UK cohort (n=262)

| **Spanish and Italian** | |  |  |  |  |  |
| --- | --- | --- | --- | --- | --- | --- |
|  |  | DQ2.2 | |  |  |  |
|  |  | + | - |  | sensitivity | 1.000 |
| SNP prediction | + | 33 | 0 | 33 | specificity | 1.000 |
|  | - | 0 | 43 | 43 | positive predictive value | 1.000 |
|  |  | 33 | 43 | 76 | r-squared | 1.000 |
|  |  |  |  |  |  |  |
|  |  | DQ2.5 | |  |  |  |
|  |  | + | - |  | sensitivity | 0.967 |
| SNP prediction | + | 29 | 3 | 32 | specificity | 0.935 |
|  | - | 1 | 43 | 44 | positive predictive value | 0.906 |
|  |  | 30 | 46 | 76 | r-squared | 0.795 |
|  |  |  |  |  |  |  |
|  |  | DQ7 | |  |  |  |
|  |  | + | - |  | sensitivity | 0.969 |
| SNP prediction | + | 31 | 1 | 32 | specificity | 0.977 |
|  | - | 1 | 43 | 44 | positive predictive value | 0.969 |
|  |  | 32 | 44 | 76 | r-squared | 0.894 |
|  |  |  |  |  |  | 0.069 |
|  |  | DQ8 | |  |  |  |
|  |  | + | - |  | sensitivity | 0.909 |
| SNP prediction | + | 10 | 0 | 10 | specificity | 1.000 |
|  | - | 1 | 65 | 66 | positive predictive value | 1.000 |
|  |  | 11 | 65 | 76 | r-squared | 0.893 |
|  |  |  |  |  |  |  |
|  |  |  |  |  |  |  |
| **UK samples** | |  |  |  |  |  |
|  |  | DQ2.5 | |  |  |  |
|  |  | + | - |  | sensitivity | 0.996 |
| SNP prediction | + | 234 | 0 | 234 | specificity | 1.000 |
|  | - | 1 | 27 | 28 | positive predictive value | 1.000 |
|  |  | 235 | 27 | 262 | r-squared | 0.960 |
